# Nonlinear reconstruction of bioclimatic outdoor-environment dynamics for the Lower Silesia region (SW Poland)

**DOI:** 10.1007/s00484-021-02101-4

**Published:** 2021-03-27

**Authors:** Arkadiusz Głogowski, Paolo Perona, Krystyna Bryś, Tadeusz Bryś

**Affiliations:** 1 Institute of Environmental Protection and Development, Faculty of Environmental Engineering and Geodesy, Wroclaw University of Environmental and Life Science, pl. Grunwaldzki 24, 50-363 Wrocław, Poland; 2 School of Engineering, The University of Edinburgh, Mayfield Road, EH93JL Edinburgh, UK; 3 Ecological Engineering Laboratory (ECOL), Institute of Environmental Sciences and Technology (IIE), ENAC Faculty, Ecole Politechnique Federale del Lausanne (EPFL), Lausanne, Switzerland; 4 Polish Geophysical Society, Wrocław Division, pl. Grunwaldzki 24, 50-357 Wrocław, Poland

**Keywords:** UTCI, Outdoor environment, Time-series, Machine learning, AMO

## Abstract

Measured meteorological time series are frequently used to obtain information about climate dynamics. We use time series analysis and nonlinear system identification methods in order to assess outdoor-environment bioclimatic conditions starting from the analysis of long historical meteorological data records. We investigate and model the stochastic and deterministic properties of 117 years (1891–2007) of monthly measurements of air temperature, precipitation and sunshine duration by separating their slow and fast components of the dynamics. In particular, we reconstruct the trend behaviour at long terms by modelling its dynamics via a phase space dynamical systems approach. The long-term reconstruction method reveals that an underlying dynamical system would drive the trend behaviour of the meteorological variables and in turn of the calculated Universal Thermal Climatic Index (UTCI), as representative of bioclimatic conditions. At longer terms, the system would slowly be attracted to a limit cycle characterized by 50–60 years cycle fluctuations that is reminiscent of the Atlantic Multidecadal Oscillation (AMO). Because of lack of information about long historical wind speed data we performed a sensitivity analysis of the UTCI to three constant wind speed scenarios (i.e. 0.5, 1 and 5 m/s). This methodology may be transferred to model bioclimatic conditions of nearby regions lacking of measured data but experiencing similar climatic conditions.

## Introduction

The study of bioclimatic conditions of the outdoor environment is a very important subject, in the first instance to understand how climate changes may affect society’s well being (Stocker et al. 2013). Bioclimatic assessment has found applications in a multitude of research areas relating the effects of climate change (Wu et al. 2019) on health and well-being (Bröde et al. 2018), epidemiology (Di Napoli et al. 2018), military (Galan and Guedes 2019), urban planning, etc. Di Napoli et al. (2018) correlated the Universal Thermal Climate Index (henceforth referred to as UTCI) and mortality after intense heat waves in Europe. Chinese tourism assessment was also explained by means of UTCI dynamics (Ge et al. 2017). In Australia, Coutts et al. (2016) quantified the variability of outdoor environment near central business centres in Melbourne. Ndetto and Matzarakis (2015) also used UTCI to asses bioclimatic conditions of the urban environment in Tanzania. Eventually, Bröde et al. (2012) studied outdoor thermal comfort in Brazil. These studies find practical application for instance to determine the attractiveness of tourist places like coastal and mountain towns or health resorts in such areas (Ge et al. 2017; Błażejczyk and Kunert 2011), as well as in ergonomics to determine working conditions in both indoor and outdoor environments (Bröde et al. 2018; Sen and Nag 2019).

Over 200 bioclimatic indexes were proposed in the last 100 years and used to analyse human body’s response to outdoor environmental conditions (de Freitas and Grigorieva 2017). Early methods to assess bioclimate conditions involved simple indexes based on a single parameter, e.g. like physical saturation deficit (Thilenius and Dorno 1925), or Wet Bulb Temperature *T*_*wb*_ (Haldane 1905). Notice how such variables describe meteorological processes rather than bioclimatic conditions. More advanced bioclimatic indexes use human biological variables (e.g. body temperature or energy (heat) exchange) in relation to actual meteorological conditions in order to derive the Physiological Equivalent Temperature (PET) (Mayer and Höppe 1987), or the UTCI (Błażejczyk et al. 2013; Jendritzky et al. 2012). Many bioclimatic indexes are commonly build up using heat exchange (led by sunshine duration), air temperature or air humidity (led by precipitation) in parallel with atmospheric pressure and wind speed (Fiala et al. 2012; Masterson and Richardson 1979; Bosford 1971). In April of 2009 The World Meteorological Organization (WMO) officially promoted the use of UTCI as the most suitable tool for determining bioclimatic conditions at the international symposium (WMO 2009). Additionally, in last decade many Polish scientists have used UTCI in different parts of Poland and they confirmed that this index is well suited for describing conditions of polish climate (Błażejczyk et al. 2010, 2012, 2013; Chabior 2011; Kuchcik et al. 2013; Okoniewska and Więcław^2013^; Nidzgorska-Lencewicz 2015; Bryś and Ojrzyńska 2016; Rozbicka and Rozbicki 2016, 2018). It is worth noticing that quantities like sunshine duration, air temperature and precipitation are among the basic meteorological variables used to characterize local and global both weather and climate conditions (Brönnimann 2015). Solar energy reaching Earth’s upper atmosphere is the direct engine of the Earth’s climate and its dynamics (Kondratyev 2013). Air circulation affects the spatial and temporal distribution of the above mentioned meteorological quantities and thus it contributes to sustain Earth’s climate system. Globally, climate conditions depend on mass and energy fluxes, which affect both nature biodiversity at meso and global scales, and outdoor bioclimatic conditions for human body at the local scale (Bryś et al. 2020).

Air temperature is widely recognised to be a fundamental proxy variable for the assessment of climate conditions and related changes (Stocker et al. 2013). It is linked to sunshine (e.g. number of sunny days) and to air mass conditions (i.e. humidity), and so indirectly to rainfalls and evaporation processes. There is also increasing evidence about the forcing role played by intensive human activity, whose effects are considered to be responsible for accelerating the rate of climatic changes (Stocker et al. 2013; Brönnimann 2015). Studying long-term air temperature evolution in relation to sunshine duration and precipitation is thus meaningful from a climatological viewpoint (Flohn 1957; Girs 1971; Bryson 1974; Groveman and Landsberg 1979). For example, Wrocław’s climate at specific locations results from the interaction between oceanic and continental air masses. Measurements at these locations provide an opportunity to better assess bioclimatic variability from observations (Kosiba 1948; Dubicka 1994). At sufficient large time scales, one would expect the link between sunshine duration, precipitation and air temperature to emerge as a slow component of the climate dynamics to which high frequency (correlated) stochastic fluctuations are superimposed as fast components. Reconstructing both components dynamics is the first step for advancing bioclimatological insights.

In this work we first extract the deterministic slow component linking precipitation, sunshine duration and temperature dynamics in a mechanistic way and then separate it from the fast component that has a high dimensional origin (i.e. eventually stochastic). Notice, that for these steps wind data records are only of minor importance as the above variables already contain the effect of air circulation. In this sense, wind speed would be a redundant variable. However, wind speed is of importance for the calculation of the UTCI where wind velocity has a clear effect on body-felt bioclimatic conditions. Analysis of the slow component shows that the dynamic is linked to Oceanic Oscillations such the North Atlantic Oscillation (NAO) or Atlantic Multidecadal Oscillation (AMO) (Marsz et al. 2019; Niedźwiedź et al. 2009; Malik et al. 2018; Knudsen et al. 2011). Malik et al. (2018) stress that oceanic oscillations like AMO, Pacific decadal oscillation (PDO) or El-Niño southern oscillation (ENSO) are mostly driven by sun activity and influence Sea Surface Temperature (SST) (Otterå et al. 2010; Peng et al. 2013; Niedzielski 2011, 2014). Using the UTCI as reference index, we show that successful signal decomposition may not only provide insights about the dynamics of the climate system at large time scale but also offer a new methodology to calculate bioclimatic indexes at long terms.

## Materials and methods

### Geographical location and data

Wrocław (SW Poland) has one of the longest measured time series of air temperature and precipitation in the World, its origins began in 1791. These series were reconstructed and homogenized from data measured over 10 different locations in Wrocław. The basis of this homogenization is from Wrocław university meteorological tower (known as Breslau Sternwarte), where measurements were taken in the period 1791–1920. Successive almost 100 years were homogenized from other 9 stations due to the numerous administrative decisions and technological improvements determining location changes. A comprehensive explanation of this homogenization may be found in the works by Bryś and Bryś (2005, 2010a). Here it suffices to say that homogenization and reconstructions techniques enabled to obtain an information about other meteorological variables like sunshine duration and water vapour pressure. Closer details of how sunshine duration time series (since 1891) were obtained may also be found in Bryś and Bryś (2003), whereas about water vapour pressure (since 1883) we refer to the work of Bryś and Bryś (2001).

**Fig. 1:**
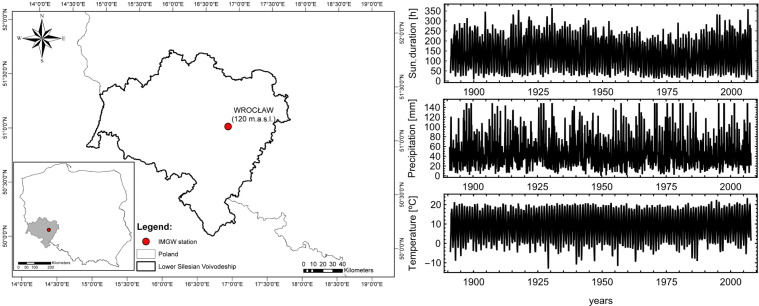
Localization of analysed area in Poland (left), with raw-data in years 1891–2007 (right)

Data used in the following work accounts for monthly sums of hours of sunshine duration (*s*) and precipitation (*p*), as well as monthly averages for air temperature (*t*) in Wrocław for period 1891–2007 (Fig. 1). The above said variables are typically used as a proxy for computing other quantities such, solar radiation (*R*), cloudiness (*N*), water vapour pressure (*e*), whence the importance of modelling them for forecasting purposes. As far as wind is concerned, this variable was extracted from the repository of the Polish Institute of Meteorology and Water Management National Research Institute https://dane.imgw.pl/ using the ‘climate’ package in R (Czernecki et al. 2020) and was only available for the 1966–2019. In general, monthly mean values for wind speed fluctuate between 0 and 4.9 m/s, being this the highest mean monthly wind speed observed in Wrocław (1966–2019). In Wrocław (1966–2019) mean monthly sum insolation oscillate between 40.7 h in December and 206 h in August with annual mean 1490.62 h. Mean monthly sum of precipitation change between 25.2 mm in February to 89.9 mm in July with annual sum 565.6 mm, Mean monthly temperature is equal − 0.1 ^∘^C in January and reach 19.7 ^∘^C in July, the mean annual value of air temperature is equal 9.6 ^∘^C

### Separation of the slow and fast signal components

All time series are clearly non-stationary because of the presence of a long-term trend affecting the data (Fig. 1). This phenomenon is typical of climatic data at all scales, and over the period of available measurements is considered an evidence of changes occurring in the Anthropocene (Brönnimann et al. 2019). Each data series was further decomposed by separating the slow component, *x*_*s*_(*t*) representing the non-linear, long-term trend from the fast component, *x*_*f*_(*t*), which represents the seasonal and the stochastic components affecting each meteorological variable (Brockwell and Davis 2016)

1 $$ x(t) = x_{s}(t) + x_{f}(t). $$

The long-term trend *x*_*s*_(*t*) was obtained by first performing a ten-years moving average with symmetric window, and then a frequency (i.e. Fourier) analysis of the resulting signals, which enhanced the dominant frequency still affecting all data series. The moving averaged data was then filtered at that identified frequency by using a low-pass Butterworth algorithm. This produced the data series *x*_*s*_(*t*) with which the original data were eventually detrended from the slow component.

The fast component, *x*_*f*_(*t*), thus resulted from removing the slow component from the original data. However, *x*_*f*_(*t*) still accounts for yearly seasonal (i.e. periodic) variability $x_{f_{p}}(t)$ affecting both the mean and the variance, and monthly correlated fluctuations $x_{f_{c}}(t)$, so that the original data was further decomposed as

2 $$ x(t) = x_{s}(t) + x_{f}(t) = x_{s}(t) + x_{f_{p}}(t) + x_{f_{c}}(t), $$

as described in the next paragraph.

### Modeling the fast component *x*_*f*_(*t*)

Seasonal fluctuations $x_{f_{s}}(t)$ were very well described by the monthly mean of the long-term detrended data, and so additionally removed from the detrended data in order to obtain $x_{f_{c}}(t)$. Notice, that this data series, although stationary, potentially still contains some temporal correlation emerging from both spatial and temporal meteorological circulation dynamics in the atmosphere. Therefore, $x_{f_{c}}(t)$ was still further decomposed into a deterministic and a stochastic fluctuation representing the intrinsic noise resulting from such complex dynamics. The time series $x_{f_{c}}(t)$ was then standardized, i.e. by subtracting the mean and by dividing by its standard

deviation, to obtain a series $x^{\prime }_{f_{c}}(t)$ having zero mean and unit variance. As the temporal correlation affecting the standardized data had almost an exponential decreasing structure, we opted to remove it by means of linear stochastic models, e.g. like the AutoRegressive AR(p) model of *p* th order (Eq. (3)) (Maidment et al. 1993; Salas et al. 1985; Haltiner and Salas 1988)

3 $$ x^{\prime}_{f_{c}}(t) = \sum\limits_{j=1}^{p} \phi_{j} (x^{\prime}_{f_{c}}(t-j)-\mu) +\epsilon_{t}, $$

with p autoregressive parameters *ϕ*(1),...,*ϕ*(*p*). The noise *𝜖*_*t*_ in Eq. (3) is an uncorrelated gaussian process with zero mean and unit variance (Maidment et al. 1993). The data was checked for the most suitable AR model order by computing the sample autocorrelation (ACF) and the partial autocorrelation functions (PACF) (Brockwell and Davis 2016), whose expressions are well known and will here be omitted. The most suitable AR model for each time series was then tested to remove any correlation in the residuals, and later used for generating statistically equivalent time series as well for forecasting purposes (Brockwell and Davis 2016).

### Modeling the slow component *x*_*s*_(*t*)

The long-term trend, *x*_*s*_(*t*) affecting each data series led us hypothesize the presence of a low-dimensional dynamics linking the three variables, sunshine duration, precipitation and air temperature at such time scales. This is motivated by the fact that sunshine duration governs soil-atmosphere heat exchange processes, evapotranspiration among which drives precipitation depending on air temperature conditions, which all feedback on sunshine duration. At large time scales, one would therefore expect that such three variables may well represent the average status of the climate of the region and can therefore be adopted as state variables of the dynamical system. In turn, this motivates the seek of a dynamical model mimicking the data that may help understanding towards which long-term dynamics the system is pointing. We therefore adopted a dynamical systems type of approach and hypothesized that such a (climate) system is currently being evolving along a non-stationary trajectory as a result of the 3-dimensional autonomous system of Ordinary Differential Equation (ODEs)

4 $$ \left\{\begin{array}{ll} \dot{x}=f_{s}(x,y,z)\\ \dot{y}=f_{p}(x,y,z)\\ \dot{z}=f_{T}(x,y,z) \end{array}\right., $$

where *x*(*t*), *y*(*t*) and *z*(*t*) are sunshine duration, precipitation, and air temperature, respectively and *t* is time. In particular, the three scalar functions were chosen in the form of a simple complete 3-order polynomial

5 $$ \left\{ \begin{array}{ll} \dot{x}=c_{1,1}+c_{1,2}x+c_{1,3}y+c_{1,4}z+...+c_{1,20}z^{3}\\ \dot{y}=c_{2,1}+c_{2,2}x+c_{2,3}y+c_{2,4}z+...+c_{2,20}z^{3}\\ \dot{z}=c_{3,1}+c_{3,2}x+c_{3,3}y+c_{3,4}z+...+c_{3,20}z^{3} \end{array} \right., $$

where *c*_*i*,*k*_ are the coefficients to be optimized. For the optimization process of such a strongly nonlinear system we appealed to a system identification techniques working in the phase space and named ‘Trajectory Method’ (Eisenhammer et al. 1991; Perona et al. 2000). This methodology compares the model performance against the observed system trajectory in the phase space *x*(*t*),*y*(*t*),*z*(*t*) and for each component builds the quality function

6 $$ Q^{i}=\sum\limits_{j=1}^{j_{max}}\sum\limits_{l=1}^{l_{max}} ||{x^{i}_{m}}(t_{j}+\triangle t_{l})-{x^{i}_{r}}(t_{j}+\triangle t_{l}))||, $$

for *j*_*max*_ initial conditions taken on the observed variable *x*_*r*_(*t*) and let the model variable *x*_*m*_(*t*) to evolve for a time *t*_*l*_ = Δ*t*2^*l*− 1^,(*l*,1..*l*_*max*_) (e.g. see Perona et al. (2000) for details). Optimum values of the coefficients *c*_*i*,*k*_ are then obtained through a minimization process of quality function (Eq. (6)) using the least-squares method (Eisenhammer et al. 1991; Perona et al. 2000). When the level of noise in the observed data is not too high, then the model would converge ideally by switching off those coefficients corresponding to the monomial terms that do not contribute to the dynamics (Perona et al. 2000). This methodology is very robust against model instabilities and has successfully been applied to model several processes in nature (Perona et al. 1998; Perona et al. 2001; Perona and Burlando 2008). We study the model dynamics from a more analytical point of view by restricting our analysis to the equilibrium points and their linear stability. By definition, equilibrium points correspond to the points in the state space where temporal derivatives of the flow nullify. Therefore, equilibrium points can be found by solving the algebraic system

7 $$ \left\{\begin{array}{ll} f_{s}(x,y,z)=0\\ f_{p}(x,y,z)=0\\ f_{T}(x,y,z)=0. \end{array}\right. $$

From a geometrical point of view, such points are found at the intersection of the curves where each flow component has null time derivative (isoclines). In order to inquire the stability of equilibrium points we performed a linear stability analysis, whose details can be found in any analytical mechanics books, e.g. see Strogatz (2018) Here it suffices to recall that the main steps of the linear stability analysis are to first linearize the model (Eq. (5)) and then to calculate the corresponding eigenvalues of the matrix of the first order partial derivatives (Jacobian matrix) at each equilibrium point. The sign (i.e. positive or negative) and the domain (i.e. real or imaginary) of the eigenvalues define the stability properties of the equilibrium point along the principal axes of the phase space.

### Universal Thermal Climate Index

Universal Thermal Climate Index (UTCI) is the common index used to assess bioclimatic conditions because of its general applicability across the year (Havenith et al. 2012). The variability of UTCI describes hot and cold human comfort responses on outdoor environment. Because of its definition, the UTCI is the most comprehensive index involving parameters such as air temperature (*T*), wind speed (*v*), water vapour pressure (*e*) and mean radiant temperature (*T*_*mrt*_) (Fiala et al. 2012),

8 $$ UTCI= f(T,v,e,T_{mrt}). $$

Water vapour pressure and mean radiant temperature can be calculated from sunshine duration, precipitation and mean air temperature, whereas wind speed is generically assumed constant and used as a sensitivity parameter. In particular, mean radiant temperature, *T*_*mrt*_, was calculated from sunshine duration by using the SolAlt formula from MENEX 2005 (Błażejczyk 2005) and implemented in the Bioklima software (Błażejczyk 1996):

9 $$ T_{mrt} = \left( \frac{\frac{R}{Irc}+0.5L_{g}+0.5L_{a}}{s_{h}\cdot\sigma}\right)^{0.25}-273. $$

In Eq. (9), *R* is absorbed solar radiation (W m^− 2^), *Irc* is the coefficient reducing convective and radiative heat transfer through clothing, *L*_*g*_ is ground radiation (W m^− 2^), *L*_*a*_ is atmosphere back radiation (W m^− 2^), *s*_*h*_ is the emissivity coefficient for humans (0.95) and *σ* is the Stefan-Boltzmann constant (5.667 ⋅ 10^− 8^ W*m*^− 2^ K^− 4^). Absorbed solar radiation (*R*) was calculated using the SolAlt model based on cloudiness (*N* [%]) and position of the Sun (hSl [^∘^]) and detailed formulas are published Błażejczyk (2005). For monthly data the position of the Sun was taken from the middle position in each month. The water vapour pressure for the data was reconstructed and homogenized following Bryś and Bryś (2010b) and modelled using Tetens’ formula (Tetens 1930).

Eventually, mean monthly values of the independent variables defining the UTCI, were all inside the range of the limiting conditions of applicability so that the UTCI could easily be computed using the Bioklima software (Havenith et al. 2012).

Our data analysis and modelling of sunshine duration, precipitation and temperature was then useful to obtain projections of such variables at long term, and then the UTCI of the region. In particular, this was done by generating synthetic data from re-aggregation of the slow and the fast components, i.e. as per Eq. (2).

In order to analyse the sensitivity of the UTCI model to wind speed conditions, we used 0.5, 1, and 5 m/s. For this wind speed scenarios we both reconstructed UTCI values for the 117 years of available data and performed long-term calculations in order to explore UTCI variability at long term.

## Results

### Signal decomposition: the slow and the fast components

**Fig. 2:**
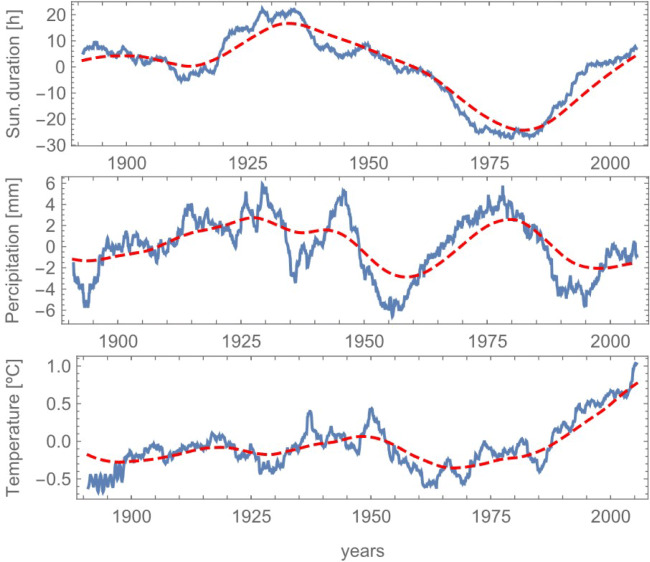
Ten years moving average (blue line) and non-linear trend (dashed red line) obtained from filtering the moving averaged data with a Butterworth low-pass filter

Figure 2 shows the long-term trends (red curves) that emerged for each time series as a result of the moving average and the Butterworth low-pass filter with a cutting frequency of 0.0038 month^− 1^. After removing the small artificial delay introduced by the moving average and the filter, the trend was adopted as slow component and removed from the original data in order to make them stationary. Whilst the trend behaviour for sunshine duration and precipitation appear fluctuating almost with zero mean, the one for temperature clearly shows a positive drift affecting the last years of the series in agreement with climate observations of ongoing changes. The relatively clear behaviour of the three series leaves also to suppose the presence of a low-dimensional dynamics underneath the data and linking such three climatic variables in a deterministic fashion. This aspect will be further addressed in ‘The slow-component as dynamical system’ ahead.

**Fig. 3:**
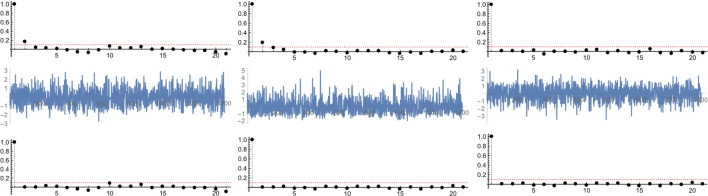
Autocorrelation function (ACF) of sunshine duration, precipitation and air temperature(upper) with residuals of Autoregresive models (AR) (middle) and test of autocorrelation by ACF of residual (bottom) for each meteorological parameter

After removing the trend, the resulting time series *x*_*f*_(*t*) was de-seasonalized and then standardized to obtain *xf*′(*t*) (Fig. 3, mid panels). Both sunshine duration and precipitation show the presence of a weak but statistically significant temporal autocorrelation, which indicates that fluctuations in the series do not have a completely random (i.e. white noise) origin, but still present a deterministic dependency on previous data back to some time lag and only statistically non-significant residual oscillations (Fig. 3 top panels). In particular, sunshine duration shows a significant correlation with previous data up to lag 1, precipitation up to lag 2 and temperature none.

The Autoregresive models (Eq. (3)) described in ‘Materials and methods’ were then used (AR(1) for sunshine duration and AR(2) for precipitation) to remove the residual correlation, thus leaving completely uncorrelated residuals (Fig. 3 lower panels). We concluded that the fast component of such climatic time series is composed of deterministic seasonal fluctuations, plus coloured noise that can easily be replicated or synthesized by means of simple linear autoregressive models.

### The slow-component as dynamical system

**Fig. 4:**
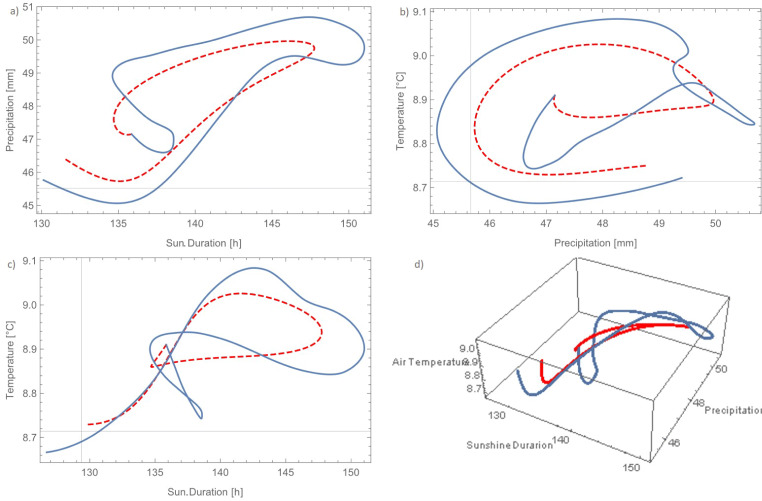
Phase space data compared to model results of precipitation and sunshine duration (**a**), air temperature precipitation (**b**), temperature and sunshine duration (**c**) and 3-dimensional phase space (**d**)

**Fig. 5:**
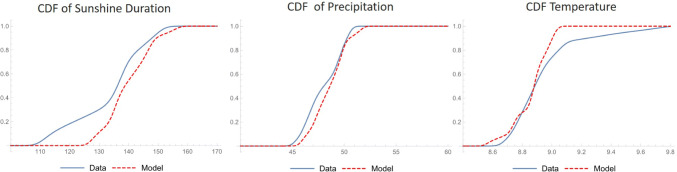
Cumulative density function (CDF) for the measured and modelled time-series of sunshine duration, precipitation and air temperature in Wrocław

The three time series forming the slow component were analysed with the Trajectory Method (‘Modeling the slow component *x*_*s*_(*t*)’) in order to seek for a low-dimensional dynamical system explaining the mechanistic structure underlying the data. When using a number of initial states, *j*_*max*_ = 48, with an inter-distance between them *d* = 7 and sequential model evolutions up to 25 data (*l*_*max*_ = 4) per each initial condition, the Trajectory Method returned a set of coefficients describing a stable 3D dynamical system that mimicked both the single time series and their trajectory in the phase space (Fig 4). It has to be stressed that the reconstruction technique was unsuccessful in the majority of the trials run by changing reconstruction parameters (3600 total trials). Only in about 17 cases the method returned a stable system and only in about 5 cases the system reproduced to some similarity the system trajectory, in some cases diverging on the long term. The parameter set presented here was the only one that represented a stable dynamical system with the minimum error function. The scarcity of meaningful models found by the method is surprising given the elevated number of coefficient involved. Despite having low physical meaning, polynomial models with such a high number of coefficients usually guarantee a high flexibility in reproducing the observed data (Perona et al. 2000). To some extent this evidences the uniqueness of the model that is able to reproduce such a complex dynamic behaviour, as shown in Fig. 4. Panels a–d show the projection of the phase space along all 2-D variable pairs as well as the 3-D phase space trajectory. The measured data are qualitatively well represented, although quantitatively some differences are clearly evident. The cumulative density functions (Fig. 5 middle) shows a very good estimation of precipitation but an overestimation of the air temperature values in the range between 8.9 and 9.1 ^∘^C (Fig. 5 right). Sunshine duration is instead underestimated in the range between 110 and 135 $\frac {\text {h}}{\text {month}}$ (Fig. 5 left).

The underestimated values of the hours of sunshine duration are strongly related with the overestimated values of air temperature in the last 20 years. This relationship has thus low physical sense, because air temperature would rise despite the less energy income from the Sun. Mutual strong relationship between all measured variables in the model will be further explained in the next section.

Despite the lack of information of pollutant, ozone’s and other parameters that are considered direct responsible of global warming (Stocker et al. 2013), the model shows a very similar behaviour to the analysed time-series in Wrocław for the last 20 years. It is therefore instructive to study its dynamical properties in order to inquire the system behaviour at long term.

### Slow component dynamical properties: long-term attractor, equilibrium points and related stability

The model of the slow component represents the dynamic behaviour of an autonomous, nonlinear and strongly dissipative system. Assuming that the real system is currently experiencing a transient dynamics undergoing climate changes, the model might provide some insights about the asymptotic behaviour of the real system under present environmental constraints. In other words, the model evolution at long term will occur towards an attractor, that is the geometrical object in the phase space representing the topological manifold of the dynamics.

**Fig. 6:**
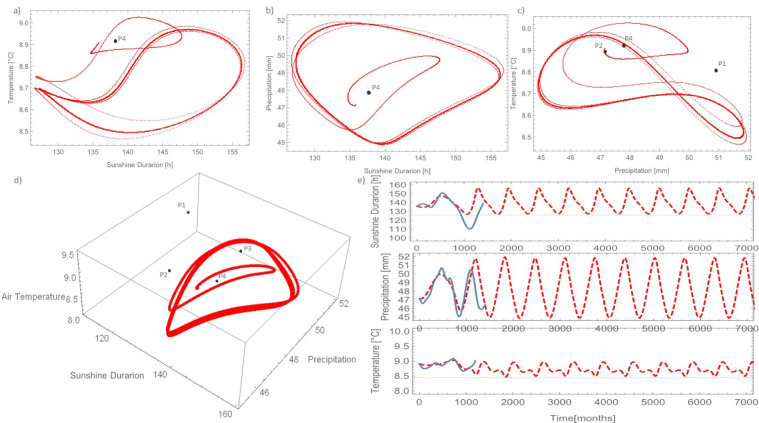
Long-term prediction for model in phase space of sunshine duration, precipitation and air temperature (**a**–**c**), 3-dimensional space with equilibrium points (**d**) and long-term courses of analyse all components with original trend (**e**)

From a purely numerical point of view, the model shows to converge onto a periodic behaviour for all variables as shown from the projection of the phase space (Fig. 6a-c). In the phase space, this results into a 3D closed trajectory that attracts the ODEs’ system when started from any initial conditions taken within the ‘so-called’ basin of attraction (Fig. 6d). To this regard, Fig. 6 show the outcome of the model after 7000 iterations, which correspond to about 583 years (117 reconstructed and 466 forecast). The length of available observations clearly belong to the initial transient phase of the model, which appears then to stabilize onto a periodic pattern after some periods (Fig. 6e). The periodicity of the oscillations is about 642 months (i.e. 53.5 years). This oscillation may be related to the Atlantic Multidecadal Oscillation (AMO), which has an approximate periodicity ranging between 55 and 80 years (see also ‘Discussion’ ahead). The stability analysis of the model shows that the model has only four real equilibrium points (‘Modeling the slow component *x*_*s*_(*t*)’ Eq. (7)), which are, for the sake of our discussion, the interesting ones (Table 1). Equilibrium points are shown in the phase space (Fig. 6d). Equilibrium points can be either stable or unstable in the sense that they either attract or repel model trajectories in the phase space, respectively. Table 1 shows that all equilibrium points have no eigenvalues with zero real part. Mathematically, this ensures that all equilibrium points are ‘hyperbolic’, that is the stability properties of the linear system are representative of those of the nonlinear one in the vicinity of the equilibrium points. Moreover, all equilibrium points have at least one eigenvalue with real positive part, which means that all equilibrium points are unstable at least along one direction.

**Table 1:** Equilibrium points and related eigenvalues defining their characteristics

Points	Equilibrium points	Eigenvalues
Sunshine duration [X_1_]	110.481	0.0164 + 0.0133i
Precipitation [Y_1_]	51.145	0.0164 − 0.0133i
Temperature [Z_1_]	8.798	− 0.0163
Sunshine duration [X_2_]	125.214	0.00044 + 0.0133i
Precipitation [Y_2_]	47.316	0.00044 − 0.0133i
Temperature [Z_2_]	8.890	0.0069
Sunshine duration [X_3_]	130.006	− 0.0115 + 0.0206i
Precipitation [Y_3_]	51.314	− 0.0115 − 0.0206i
Temperature [Z_3_]	8.378	0.0224
Sunshine Duration [X_4_]	138.049	0.001821 + 0.00776i
Precipitation [Y_4_]	47.908	0.00182 − 0.00776i
Temperature [Z_4_]	8.923	− 0.00541
Points		Characteristic
P_1_(*X*_1_,*Y*_1_,*Z*_1_)		unstable focus in (X,Y), stable node along Z
P_2_(*X*_2_,*Y*_2_,*Z*_2_)		unstable focus in (X,Y), unstable node along Z
P_3_(*X*_3_,*Y*_3_,*Z*_3_)		stable focus in (X,Y), unstable node along Z
P_4_(*X*_4_,*Y*_4_,*Z*_4_)		unstable focus in (X,Y), stable node along Z

In our model *P*_1_ and *P*_4_ behave as unstable focus in the (X,Y) plane, and as stable nodes along the Z direction. Thus, trajectories spiral away from the equilibrium point in the X,Y plane, whereas are straightly attracted towards the point along Z. Equilibrium point *P*_2_ is instead unstable in all directions. Next to point *P*_3_ trajectories will spiral towards the point in the X,Y plane and be straightly repelled along Z. Overall, we can therefore conclude that the system is locally unstable in the vicinity of the equilibrium points where trajectory would sooner or later drift away from the points. However, as we verified numerically, the system is globally stable because of the presence of the limit cycle, which is an actual invariant manifold of the system and result from the interplay between attraction and repellion of the equilibrium point along the three coordinate axes X,Y,Z. The stability properies of the limit cycle could be well investigated by mean of Floquet theory (Strogatz 2018), which is however not object of the present work. The significance and implications for the slow component of the system dynamical properties presented above will be discussed in the next section.

### Reconstruction and prediction of bioclimatic conditions of the outdoor environment

**Fig. 7:**
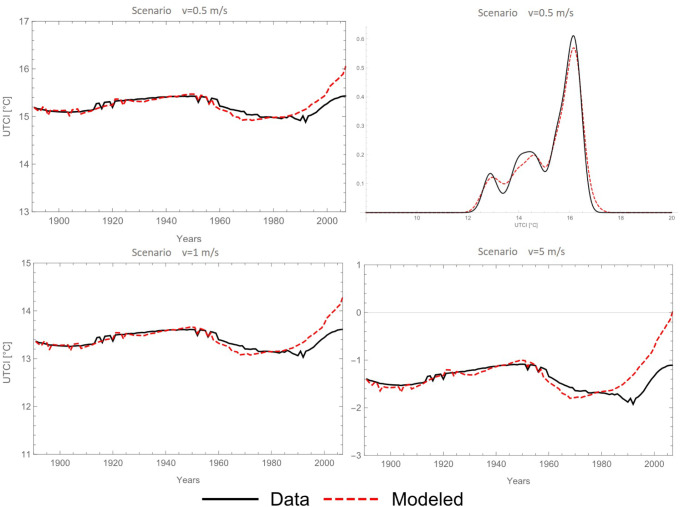
Monthly UTCI values for 0.5 m/s scenario with comparison of probability density functions (upper panels) with UTCI in two other scenarios (1, 5 m/s) for data and modelled slow component time series

Assessment of outdoor environment for both the actual and modelled case was made by means of the UTCI, which was calculated as explained in ‘Materials and methods’. UTCI was calculated from non-linear slow component obtained from the data or from the modelled slow component. Values of UTCI monthly data oscillate in a range from − 15 to 30 ^∘^C and both UTCI (i.e. from data or from modelled data) agree very well in average, which reaches about 15.2 ^∘^C UTCI (Fig. 7 upper left). Changes of UTCI calculated from slow components are in the range between 10.5 and 15 ^∘^C UTCI. The mean square error (MSE) between modelled and obtained slow component from data is equal 0.076 what was also identified on a comparison of those time series at probability density function (Fig. 7, upper right). Comparison of 1 m/s and 5 m/s approaches was presented on the bottom panels of Fig. 7. By increasing the wind speed the difference in compatibility between reconstructed and real data increases. MSE on 5 m/s scenario increase to 0.15.

The differences between the subtracted and modelled slow component in assessment of the UTCI significantly change. The most impacted element in shaping UTCI values is air temperature. The worst predicted temperature, with maximum error about 0.2 ^∘^C (Fig. 6), caused big changes in sunshine duration and precipitation mostly because scale of units. This change is less relevant in the bioclimatic outdoor environmental assessment then in global climate changes, where air temperature is also the most responsive parameter. This small variance of air temperature in the model may have application in reconstructing and forecasting the bioclimatic outdoor environmental conditions. Despite the annual average the long-term oscillation is still visible. In the analysed period 1891–2007 one sees that two minimum values occur in a range of 60–80 years. In order to better enhance the periodicity only one wind scenario was used for the forecast of the UTCI (0.5 m/s).

**Fig. 8:**
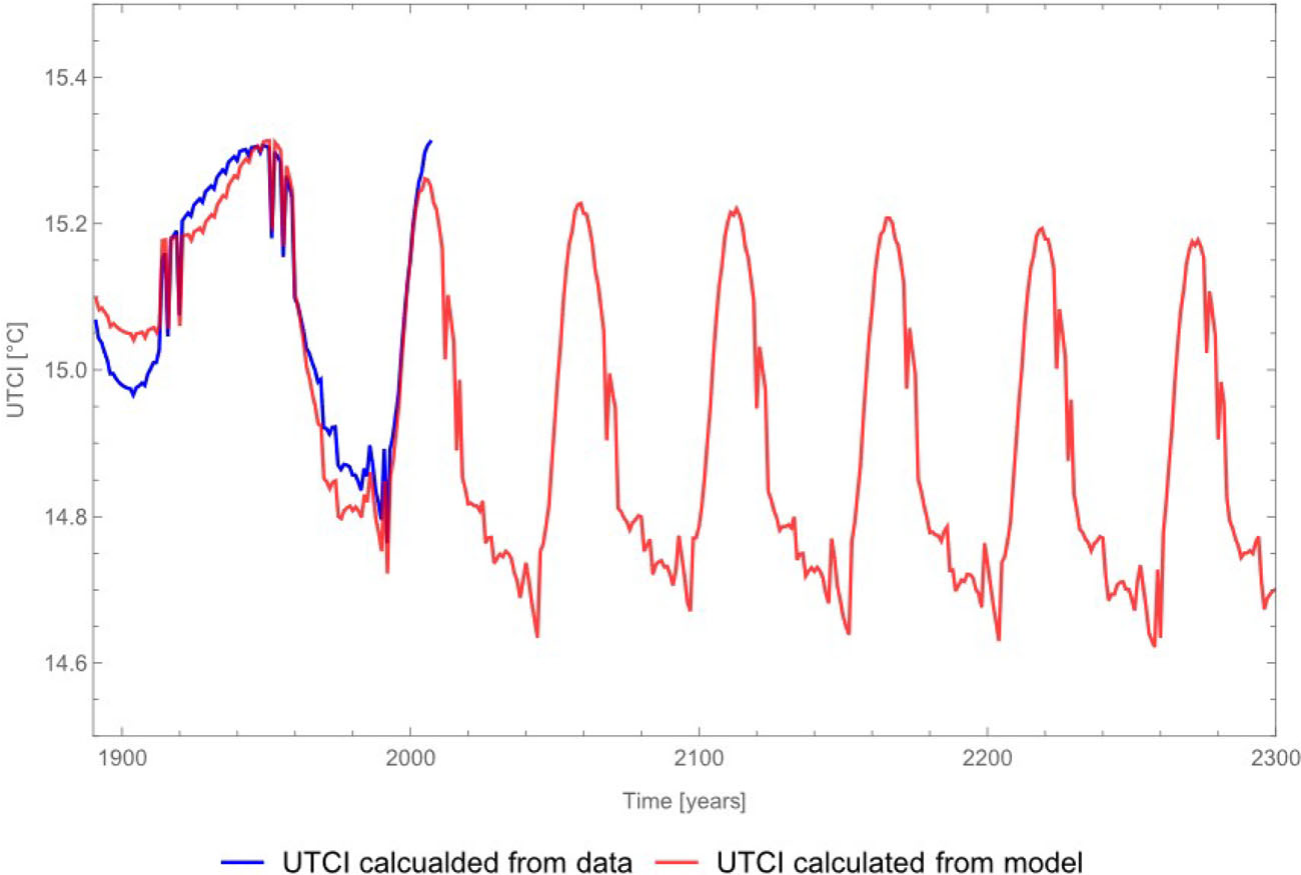
Long-term prediction of decal variability of UTCI trend compared to data obtained from slow component time series for the 0.5 m/s wind speed scenario

Figure 8 shows the comparison of the UTCI computed from the modelled slow component and the observed one for lowest wind-speed scenario. Both UTCI show similar behaviour and the modelled one allow for a long-term forecast of the underlying trend. According to the dynamics of the slow component an oscillating trend will establish under the assumption that present boundary (atmospheric and environmental) conditions do not change.

## Discussion

Stochastic and deterministic approaches have been used to model environmental processes (Perona and Burlando 2008; Chalfen et al. 2014; Czernecki et al. 2018; Malik et al. 2018), financing (Campbell et al. 1997), city transport (Kazak et al. 2017), quality assessment (DeLone and McLean 1992) and many others processes. Time series analysis of measured data may reveal the presence of low-dimensional deterministic behaviour in the slow component (e.g. the long-term trend). The corresponding dynamical system can sometimes be reconstructed starting from observations (Baake et al. 1992; Eisenhammer et al. 1991; Judd and Mees 1995; Irving and Dewson 1997; Perona et al. 2000; Perona et al. 2001). Most of such techniques are today included in machine learning approaches (Czernecki et al. 2018; Pilguj et al. 2019; Szymanowski et al. 2019).

The effort performed here to separate all components and reconstruct their physical nature has shown that the fast component has the properties of a coloured noise possibly overlapped to a seasonal behaviour. The slow component may often hinder a dynamics with a more deterministic structure. Indeed, the three variables analysed here are representative of the average state of the climatic system, and can therefore be treated as actual state variables responsible for the slow component. In other words, the state variables could be linked in a mechanistic fashion. In this study, the slow component shows a long-term oscillation that made appealing the use of reconstruction techniques. Notably, the periodic oscillation that characterizes the dynamic of the reconstructed slow component would be consistent with well known patterns induced by the Atlantic Multidecadal Oscillation (AMO) of 55–80 years (Knudsen et al. 2011; Brönnimann 2015; Malik et al. 2018). Also, Malik et al. (2018) found statistical evidence that Atlantic Multidecadal Oscillation (AMO) has intrinsic positive correlation with solar activity. Thus, after a transitory time that started in the antrhopocene, the model of the slow dynamics suggests that the system will set on to a periodic oscillation yet driven by AMO, but characterized by an offset average with respect to pre-anthropic conditions. The effect of anthropic actions are indeed implicitly appearing in the behaviour of the observed variables, which is why we adopt a dynamical system approach that objectively aims at reconstructing the dynamics. Our model for the long-term trend component describes an autonomous dynamical system (i.e. a surrogate climatic system) out of equilibrium that shows a transient behaviour leading it to future periodic oscillations different from the past ones. Hence, the model does not show who is responsible for causing such a new trajectory, only it models that there is one and that this will lead to a new periodic equilibrium.

The variability of the UTCI describing outdoor environment shows a systematic change in the duration of the oscillation over the period where observations are available. Similarly, difference from minimum and maximum values drastically increased as well as the time of these extreme periods (Fig. 8). Colder periods with average UTCI temperature lower than 15 ^∘^C lasted for about 40 years and after which UTCI values drastically grew at the beginnings of 80s. This is an effect of increased air temperature and sunshine duration which resulted in less frequent cloud cover (‘Slow component dynamical properties: long-term attractor, equilibrium points and related stability’). Decreasing of UTCI in present years is mainly caused by less frequent precipitation periods that determine air water content. In terms of bioclimatic outdoor environment, the Wroclaw case (central Europe climate) shows increasing air temperature and hours of sunshine duration, decrease a heat sensations of human body because of less humidity in the air. This situation might of course be different in other parts of the words where air humidity is already low, and air temperature is at different level then in Wrocław (central Europe).

At longer term, the model suggests that the oscillating trend will set to a constant periodicity, and generically lower mean. This is an effect of air temperature and sunshine duration form the model. According to Fig. 6 reconstructed air temperature change in bigger oscillation (having also lower values about 0.1 ^∘^C) than data. Reconstructed precipitation data oscillate in bigger range then raw data. In the other hand sunshine duration is overestimated. These small differences indicate those changes in UTCI. This was also visible in Głogowski et al. (2020) about the bioclimatic conditions of the Lower Silesia where UTCI was almost constant despite increasing air temperature. Bioclimatic conditions are ‘sum’ of all outdoor environmental conditions. The changes in air temperature may be overtaken by other factors like humidity, solar radiation or wind speed. In this case, the model overestimates sunshine duration and underestimates air temperature. The amount of precipitation was reconstructed with the best correspondence to raw data.

As stated in the introduction, UTCI found broad application to describe outdoor environmental conditions in many parts of the worlds, and may help sustaining the econmic growth of low-income countries (Sen and Nag 2019). The technique developed in this work may be useful either for reconstructing past and future UTCI dynamics or to simply generate synthetic data for filling data gaps or for statistical analyses. Another potential application could be downscaling data to regions that do not possess meteorological observations.

Perhaps in a speculative way, we attempt an interpretation of our model results keeping in mind that our model is only indirectly physically based because it entirely builds on the (nonlinear) information contained in observed data. As such the autonomous dynamical system that was reconstructed for the slow component does not include the effect of further forcing on the state variables. Under these premises, the data trend dynamics would suggest that present conditions would actually sit on a transient climatic trajectory, which will lead outdoor environmental conditions for the region to settle on a periodic long-term behaviour for the trend. The persistent increase of sunshine duration and temperature averages will eventually feedback on precipitation. Future years may then experience a precipitation increase and consequent decrease of sunshine duration and temperature with some phase delay (already present in the observations) probably due to thermal inertia of air and water masses. Hence, current transient conditions clearly emerging from observations were likely triggered by past stress on the global climate caused by severe anthropic activities. This seems however to not have altered the future footprint of multidecadal oscillations (e.g. AMO in particular) on the proxy climatic variables investigated here.

## Conclusions

A non-linear approach (trajectory method) for reconstructing ordinary differential equations from data was used in this paper in order to model the slow component affecting monthly sunshine duration, precipitation and temperature data. The reconstructed dynamical system was then used to build the aggregated UTCI representing bioclimatic conditions of the outdoor environment for the region of interest. Past and present evolution of UTCI seems to settle on long-term 50–60 years’ fluctuations where an as small as 0.1 ^∘^C change of monthly air temperature may induce changes of monthly sum of precipitation of about 5 mm, in turn causing 20 hours monthly difference in sunshine duration. Globally, this behaviour was investigated in terms of UTCI, which is however strongly dependent on wind speed as a parameter. The mean square error for low wind speed (0.5 m/s monthly value) values was very low and equal to 0.07 and for high wind speed (5 m/s monthly value) increase to 0.15. Our modelling approach is general and can be applied to any environment provided that long enough time series of measured data are available.
